# NRAS and BRAF Mutations in Melanoma-Associated Nevi and Uninvolved Nevi

**DOI:** 10.1371/journal.pone.0069639

**Published:** 2013-07-08

**Authors:** Philipp Tschandl, Anna Sophie Berghoff, Matthias Preusser, Sebastian Burgstaller-Muehlbacher, Hubert Pehamberger, Ichiro Okamoto, Harald Kittler

**Affiliations:** 1 Department of Dermatology, Medical University of Vienna, Vienna, Austria; 2 Institute of Neurology and Comprehensive Cancer Center, Medical University of Vienna, Vienna, Austria; 3 Department of Medicine I and Comprehensive Cancer Center, Medical University of Vienna, Vienna, Austria

## Abstract

According to the prevailing multistep model of melanoma development, oncogenic BRAF or NRAS mutations are crucial initial events in melanoma development. It is not known whether melanocytic nevi that are found in association with a melanoma are more likely to carry BRAF or NRAS mutations than uninvolved nevi. By laser microdissection we were able to selectively dissect and genotype cells either from the nevus or from the melanoma part of 46 melanomas that developed in association with a nevus. In 25 cases we also genotyped a control nevus of the same patients. Available tissue was also immunostained using the BRAF^V600E^-mutation specific antibody VE1. The BRAF^V600E^ mutation was found in 63.0% of melanomas, 65.2% of associated nevi and 50.0% of control nevi. No significant differences in the distribution of BRAF or NRAS mutations could be found between melanoma and associated nevi or between melanoma associated nevi and control nevi. In concordant cases immunohistochemistry showed a higher expression (intensity of immunohistochemistry) of the mutated BRAF^V600E^-protein in melanomas compared to their associated nevi. In this series the presence of a BRAF- or NRAS mutation in a nevus was not associated with the risk of malignant transformation. Our findings do not support the current traditional model of stepwise tumor progression.

## Introduction

At present, the initial genetic changes in the development of cutaneous melanoma are unclear. Our understanding of the genetic basis of melanoma development and progression is based primarily on the classic multi-step model predicting that the acquisition of oncogenic mutations is a founder event in melanocytic neoplasia. The Clark model of melanoma progression is based on the concept of a sequential accumulation of mutations that is mirrored morphologically by the transformation of a benign melanocytic nevus to a dysplastic nevus and finally to a melanoma [1–5]. At a molecular level it is believed that activation of the mitogen-activated protein kinase (MAPK) signaling pathway as a result of somatic mutations of NRAS or BRAF is a crucial event in this multistep development of melanoma [6–8]. These mutations, which occur mutually exclusive [9,10], cause constitutive activation of the serine–threonine kinases in the ERK–MAPK pathway. The role of BRAF-mutations is underlined by advances in the treatment of melanoma with BRAF inhibitors [11–13] but the exact role of BRAF in the initiation or progression of melanoma is still unknown. There are conflicting results with regard to the role of BRAF and NRAS mutations in melanomas in their horizontal and vertical growth phase [14–18]. It is also known that BRAF mutations occur at a similar frequency in nevi and in primary and metastatic melanomas [9,19–22]. It has been proposed that activating BRAF mutations induce senescence/apoptosis by up-regulating the tumor suppressor IGFBP7, which acts through autocrine/paracrine pathways to inhibit BRAF-MEK-ERK signaling. Wajapeyee and coworkers suggest that loss of IGFBP7 expression acts as a critical step in melanoma genesis [23]. Decarlo et al. on the other hand found a disparate expression of IGFBP7 in BRAF^V600E^-positive dysplastic nevi (enhanced in 56% and diminished/absent in 44%) indicating that the behavior of oncogenic BRAF in dysplastic nevi, unlike that in malignant melanoma, does not appear to consistently induce senescence/apoptosis through pathways mediated by IGFBP7 [24–26]. Most nevi, including so called “dysplastic nevi”, cease proliferation and remain static for decades. If nevi are indeed precursor lesions of melanoma they must acquire genetic alterations to free themselves of growth restraints and become malignant. The fact that oncogenic BRAF mutations are frequent in “dysplastic nevi”, congenital nevi, common nevi and especially in growing nevi [20,27,28] has challenged the role of BRAF mutations for the development of melanoma [16] in particular and the model of stepwise tumor progression in general. According to this model the “dysplastic” or “atypical nevus” is the “missing link” between a benign and a malignant melanocytic lesion [4] and should be typified by genetic alterations that differ from “common nevi” and from melanoma. The major inherent problem is the lack of interobserver agreement for the morphology-based diagnosis of “dysplastic nevi” including clinical, dermatoscopic and histopathologic diagnosis [29]. Until now, BRAF or NRAS mutations have been investigated systematically only in nevi that were not associated with melanomas [7,10,20,22,30,31]. Therefore it is unknown if BRAF or NRAS mutations play any role in the progression of a nevus into a melanoma. For a better understanding it is mandatory to study a subset of melanomas that arose in association with a preexisting nevus [32,33]. To gain a more profound understanding in the role of BRAF or NRAS mutations in the development of melanoma from nevi we compared the genotype and BRAF^V600E^ protein expression of melanomas and their associated nevi with control nevi of the same patient.

## Results

### Patient characteristics

We included 46 melanomas (from 45 patients) that developed in association with a preexisting nevus. Mean age of the patients at diagnosis was 51.4 years (SD ±15.5), 34.8% were female. In 25 patients a suitable control-nevus of the same patient was also available for genetic analysis. The majority of melanomas (74.2%) had a Breslow thickness <1.00mm (median 0.5mm, 25^th^-75^th^ percentile: 0.35-1.05mm) and 93.5% were of tumor stage T1a (n=38) or T2a (n=5). All melanomas were of superficial spreading subtype, two melanomas showed ulceration histologically. Most melanomas were located on the trunk (n=35, 76.1%), followed by the upper extremities (n=6, 13.0%), the head, neck and face (n=3, 6.5%), lower extremities (n=1, 2.2%) and acral sites (n=1, 2.2%). No mucosal melanomas were included in this study.

### Frequency of BRAF/NRAS mutations detected by Sanger sequencing

NRAS-mutations within Exon 2 were found in 11.9% (n=5), 18.2% (n=8) and 14.3% (n=3) of melanomas, associated nevi and control nevi, respectively. All non-silent mutations were substitutions of the Codon 61 (Q61K, Q61L or Q61R; Table 1).

**Table 1:** Mutations detected in samples.

**BRAF^V600^ (Sanger)**	**Melanoma (n=45)**	**Associated nevus (n=46)**	**Control nevus (n=25)**	**Matched melanoma (n=28)**
V600E	51.1% (n=23)	63.0% (n=29)	52.0% (n=13)	39.3% (n=11)
V600K	0	0	4.0% (n=1)	0
Wildtype	48.9% (n=22)	37.0% (n=17)	44.0% (n=11)	60.7% (n=17)
**BRAF^V600E^ (Sanger + VE1 IHC)**	**Melanoma (n=46)**	**Associated nevus (n=46)**	**Control nevus (n=25)**	**Matched melanoma (n=29)**
V600E	63.0% (n=29)	65.2% (n=30)	54.2% (n=13)	41.4% (n=12)
Wildtype	37.0% (n=17)	34.8% (n=16)	48.0% (n=12)	58.6% (n=17)
**NRAS Exon 2 (Sanger)**	**Melanoma (n=42)**	**Associated nevus (n=44)**	**Control nevus (n=21)**	**Matched melanoma (n=26)**
Silent mutations	2.4% (n=1; A66A)	2.3% (n=1; L52L)	0	0
Q61K	4.8% (n=2)	4.5% (n=2)	14.3% (n=3)	0
Q61L	2.4% (n=1)	2.3% (n=1)	0	0
Q61R	2.4% (n=1)	9.1% (n=4)	0	7.7% (n=2)
Wildtype	88.1% (n=37)	81.8% (n=36)	85.7% (n=18)	92.3% (n=24)

BRAF^V600^-mutations within exon 15 detected by Sanger-sequencing were present in 51.1% (n=23), 63.0% (n=29) and 52.0% (n=13) in melanomas, associated nevi and control nevi, respectively. With the exception of a single V600K substitution in a control nevus, all mutations within Codon 600 were an exchange of Valine by Glutamine (V600E) (Table 1). Four mutations in four different patients outside Codon 600 were detected: S602F (melanoma), S607F (associated nevus), R603Q (control nevus) and one single nucleotide variant within Intron 14 (dbSNP-Reference: rs143181039; melanoma, associated nevus and control nevus of one patient).

### Immunohistochemical detection of BRAF^V600E^

**Figure 1:**
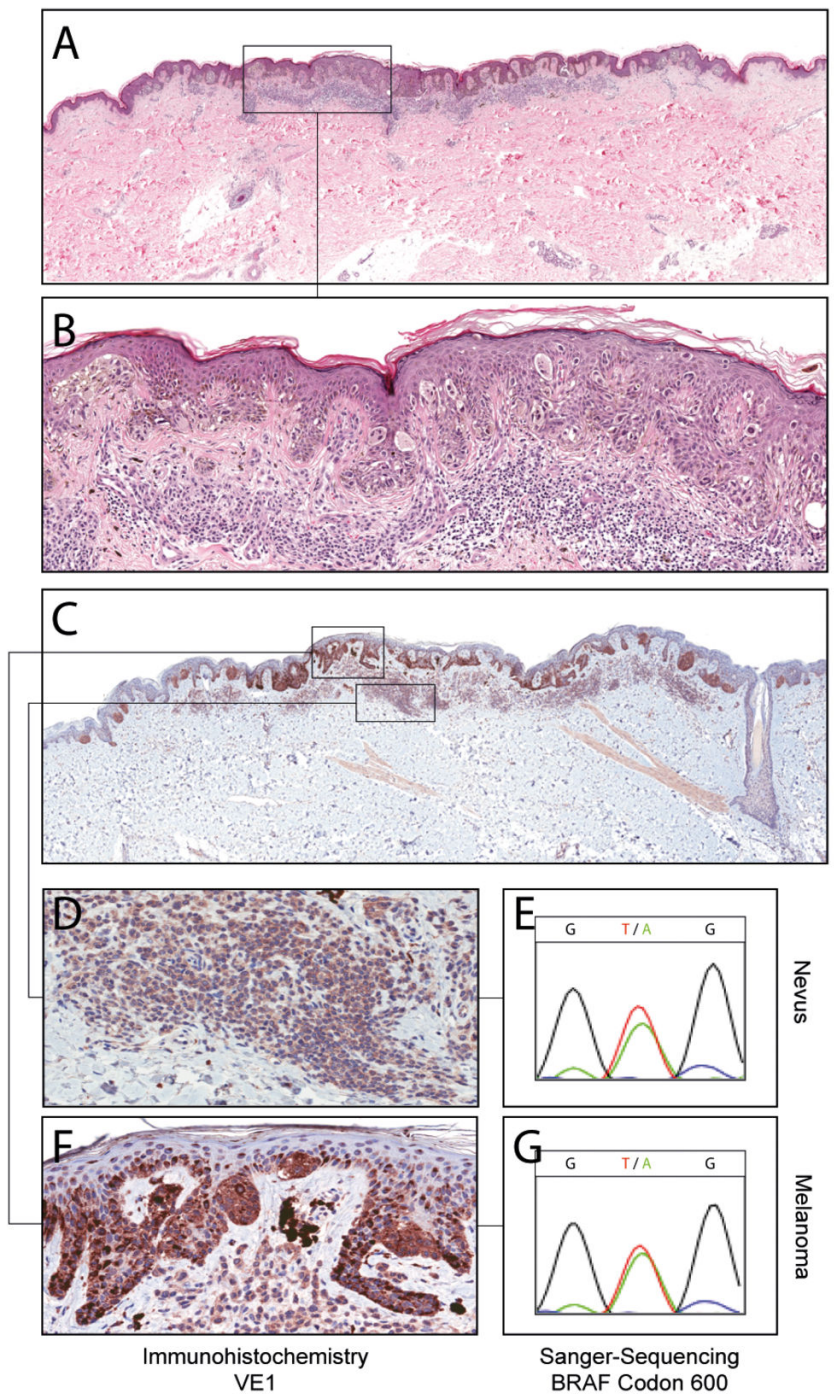
Melanoma in association with a nevus in overview (A) and closeup (B). Immunohistochemically stained in (C) with the BRAF^V600E^-mutation specific antibody VE1. Benign(D) and malignant(F) parts of the tumor in closeup. E&G show sequencing result of the corresponding cuts.

Twenty-eight melanomas and 15 control nevi were stained with the BRAF^V600E^-mutation specific antibody VE1. Immunohistochemistry detected all mutations found by Sanger sequencing (Figure 1 D–G). In 6 (5.1%) of 117 tumor-samples (5 melanomas and 1 associated nevus) immunohistochemistry showed a BRAF^V600E^ mutation not detectable by sequencing. These cases remained discordant after repeated microdissection, amplification and sequencing. While no associated nevus showed strong staining, 10.0% (n=2) of melanomas were strongly positive by immunohistochemistry. The majority of associated nevi (80.0%, n=16) stained weakly as did only 25.0% (n=5) of melanomas. Twenty percent (n=4) of associated nevi and 65.0% (n=13) of melanomas showed an intermediate staining intensity. Paired analysis revealed that melanomas showed a stronger immunohistochemical staining intensity than their associated nevi (Wilcoxon signed-rank test, p=0.002) (Figure 1 C,D,F and Figures S2 & S3 H,I).

### BRAF and NRAS mutations in control nevi, melanomas and associated nevi

**Figure 2:**
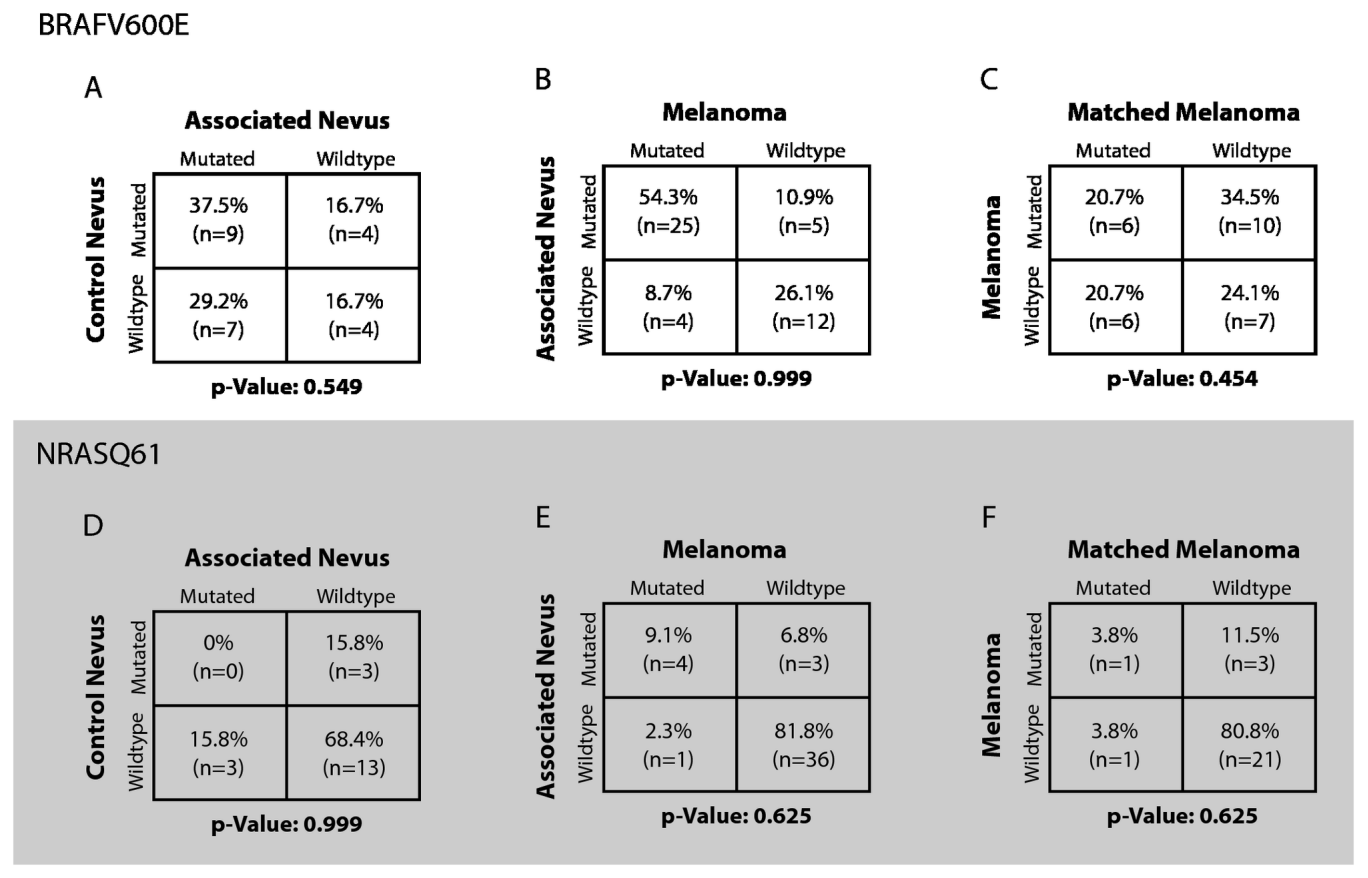
Comparison of mutation status between paired tumor groups. BRAF^V600E^-mutation status was evaluated by VE1-Immunohistochemistry and Sanger sequencing, NRAS^Q61^ by Sanger sequencing alone. p-values denote two-tailed significance as measured by McNemar test.

The frequency of oncogenic mutations did not differ significantly between melanoma-associated nevi and control-nevi or between melanomas and their associated nevi (Figures 2 and S4). However, in four pairs a BRAF-wildtype nevus was present next to a BRAF^V600E^-mutant melanoma; in five cases a BRAF-wildtype melanoma had a preexisting BRAF^V600E^-mutated nevus. BRAF^V600E^-mutation status was associated with younger age (55.3 years versus 48.2 years, p=0.007). In the present series we found no association between BRAF mutations and gender, invasion thickness or anatomic site. NRAS^Q61^ mutations were more frequently found in lesions occurring on the lower (18.2%, n=2) and upper extremities (27.8%, n=5) and less common on the trunk (7.8%, n=6).

NRAS mutations were not distributed differently between melanomas, associated nevi and control nevi. Mutation status of control nevi and melanoma-associated nevi was concordant in 68.4% (p=0.99), melanomas and preexisting nevi were concordant in 90.9% (p=0.63) (Figure 2). NRAS^Q61^-mutation status was not associated with a age, gender or invasion thickness.

### BRAF and NRAS mutations of matched melanomas

For 28 cases (26 for NRAS) a melanoma without an associated nevus, could be matched for patient age ± 3 years, gender and anatomic site. Matched melanomas harbored a BRAF^V600E^ mutation in 42.9% (n=12) of cases and NRAS^Q61^ mutations in 7.7% (n=2). The frequency of V600E or Q61 mutations was not different compared to melanomas with associated nevi (p=0.45 and p=0.63 respectively).

### Phenotype of nevi and phenotype-genotype correlation

**Figure 3:**
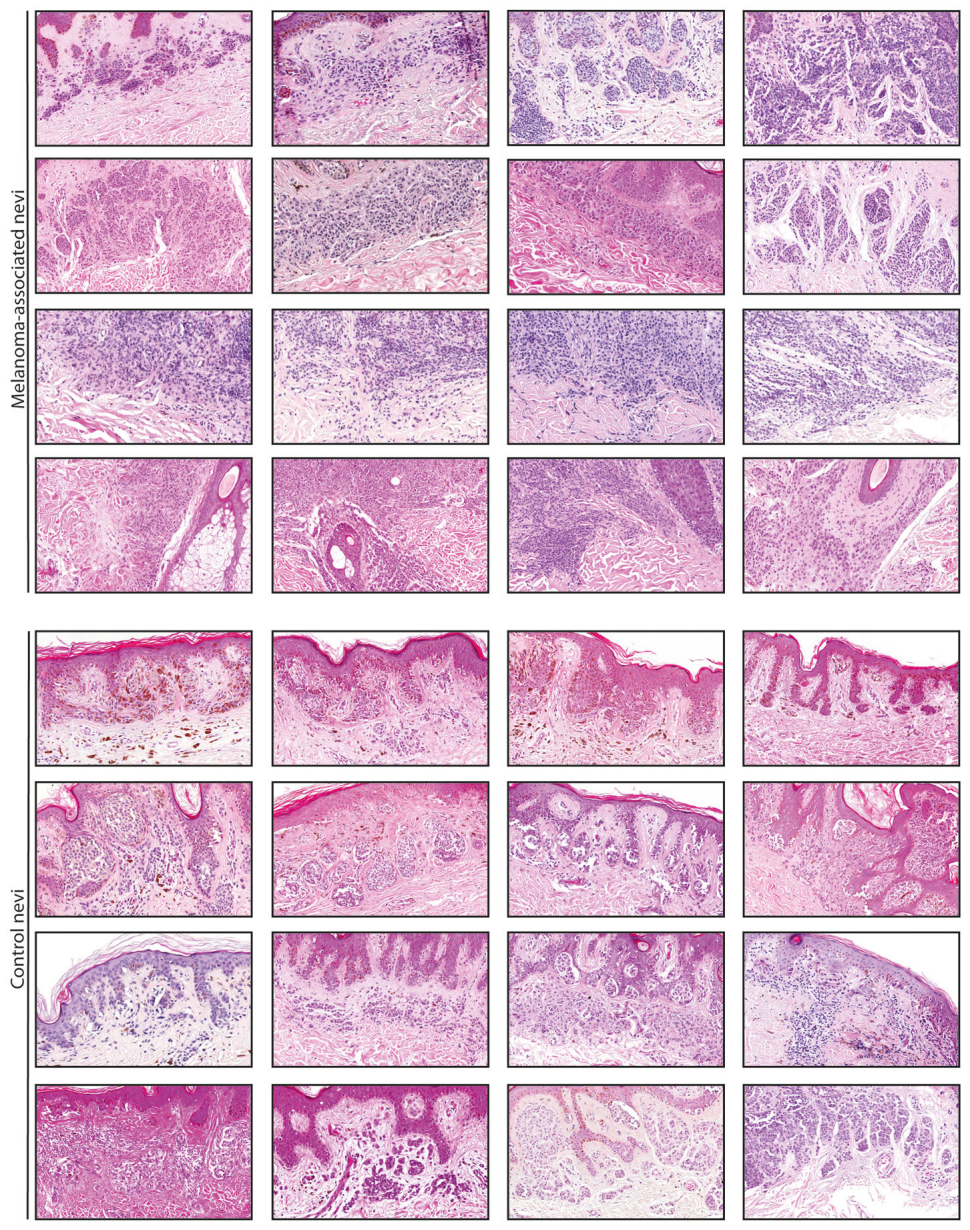
Representative tumor parts of nevi groups. Melanoma associated nevi are more commonly strictly dermal and show less of the postulated features of so called "dysplastic" nevi.

Melanoma associated nevi were either strictly dermal (84.8%) or compound nevi (15.2%). Control nevi were more likely to have a junctional component than associated nevi (92.0% versus 15.2%, p<0.001). Histomorphologic features of “dysplastic nevi” [1,34–37] (lentiginous/epitheloid cell proliferation, fibroplasia and cellular atypia) were more common in control nevi than in melanoma associated nevi (Table 2 and Figure 3). Overall, BRAF^V600E^ positive nevi were more common to have no junctional component (p=0.04) and less common to show lentiginous/epitheloid cell proliferation (p=0.023). No statistically significant correlation of other aforementioned histomorphologic features could be found with mutations within BRAF^V600^ or NRAS^Q61^.

**Table 2:** Comparison of clinical and morphologic criteria between nevi groups.

		**Associated Nevus (n=46)**	**Control Nevus (n=25)**	**p-value (Chi-square)**
**Morphology**	**Bridging**	2.2% (n=1)	20.0% (n=5)	0.018
	**Lentiginous / epitheloid cell proliferation**	10.9% (n=5)	68.0% (n=17)	**<0.001**
	**Fibroplasia**	4.3% (n=2)	52.0% (n=13)	**<0.001**
	**Cytologic atypia**	13.0% (n=6)	40.0% (n=10)	**0.009**
**Nevus subtype**	**Junctional component**	15.2% (n=7)	92.0% (n=23)	**<0.001**
**Mutations**	**NRAS^Q61^**	15.9% (n=7)	14.3% (n=3)	1.000
	**BRAF^V600^ (Seq & VE1)**	65.2% (n=30)	52.0% (n=13)	0.217
**Anatomic Site**	**Trunk**	76.1 % (n=35)	56.1% (n=14)	0.080

Because nevus type (junctional component present or not) and histomorphologic criteria were strongly correlated we built two different multivariate models to avoid collinearity. One model included nevus type the other histomorphologic criteria. If nevus type was excluded, location on the trunk (OR 6.68, SD:1.19-37.65, p=0.031) was predictive of melanoma associated nevi. Lentiginous/epitheloid cell proliferation (OR 0.06, SD:0.01-0.35, p=0.002) and fibroplasia (OR 0.12, SD:0.02-0.80, p=0.028) were significantly associated with uninvolved nevi. Using a multivariate logistic regression model excluding histomorphologic features only location on the trunk (OR 3.09, SD:1.02-9.37, p=0.046) remained a statistically significant predictor of association with melanoma.

## Discussion

The major finding of our study is that in contrast to current belief, neither morphology nor the presence of BRAF or NRAS mutation predicts the chance of malignant transformation of a nevus into a melanoma. The frequencies of oncogenic BRAF mutations were similar in the melanoma part (63.0%) and in the nevus part (65.2%) with a concordance of 80.4%. BRAF or NRAS mutated nevi did not have a higher chance to be associated with melanomas than wild type nevi. In other words, the presence of a BRAF^V600^-or NRAS^Q61^-mutation within a nevus does not change its risk of transforming into a melanoma. Our results do not support the concept that oncogenic BRAF or NRAS mutations play a major role in the development of melanoma from nevi and do not support the multistep theory of melanoma progression in its current form. Even if the multistep theory is true it is unlikely that the multiple steps of tumor progression correspond to a morphologic spectrum that spans from a benign nevus via a dysplastic nevus to melanoma in situ. It seems that if melanomas develop in a preexisting nevus it is most likely an inconspicuous nevus that shows even less histomorphologic “dysplasia” or “atypia” than a control nevus of the same patient. From a histomorphologic point of view control nevi were even more “atypical“ than those nevi associated with melanomas. However, it has to be stated that most control nevi were excised for diagnostic reasons, which would explain why they showed a higher frequency of atypical features histomorphologically. This limitation however does not attenuate the fact that the genotypes of melanoma associated nevi and control nevi were the same. It was discovered recently, that melanomas show heterogeneity in regard to BRAF-genotype [38]. It is therefore possible that the melanoma arose in melanocytes that were not sampled. Our method of genotyping - namely conventional Sanger sequencing - has been shown to have a low sensitivity in detecting BRAF^V600E^ mutations [39]. For this reason we also used immunohistochemistry, a method that has an excellent accuracy in the detection of oncogenic BRAF^V600E^ mutations and has been shown to be particularly advantageous in specimens containing only small tumor parts [40]. Addition of immunohistochemistry data did not change the significance of our results and it is therefore unlikely that our results can be explained by sampling error. Another reason for discordant melanoma-nevus pairs might be that the melanoma might have had its’ origin not in the nevus but developed from melanocytes within the epidermis having a different genotype than the nevus. Given the high concordance of BRAF and NRAF mutations in melanoma and their associated nevi we consider this possibility highly unlikely. Finally we cannot rule out completely that epidermal parts of the associated nevus have been overgrown by the melanoma and therefore have not been accessible for analysis.

Evaluating tumors with BRAF^V600E^-genotype in benign and malignant parts, the melanoma showed a more intense staining with the VE1-antibody than the associated nevus (Figure 1 C,D,F and Figures S2 & S3 H,I). This higher expression of the oncogenic protein might be due to additional genetic or epigenetic events that play a role in oncogene-induced senescence or upregulate the RAS-RAF-pathway. It is interesting that most melanomas arose in the epidermis although most associated nevi were compound. This may be explained by the fact that driver mutations that are detected in addition to BRAF-mutants show a signature of UV-mutagenesis [41].

In our series, melanomas associated with nevi showed a similar frequency of BRAF^V600^-and NRAS^Q61^-mutations compared to published reports of melanomas of the skin in general [7]. Higher mutation-rates in melanomas of younger individuals are in line with recent findings [41] that V600E mutations might not be related to chronic sun-exposure. The frequent occurrence of V600E mutations in other neoplasms such as thyroid carcinomas [42], ovarian cancer or adenocarcinomas [43] make it unlikely that actinic damage is a major cause of this mutation. A reason for more frequent BRAF^V600E^-positive melanomas in younger patients might be that in older patients a higher percentage of melanocytic lesions harboring the mutation already have undergone oncogene driven senescence [44]. When using a more sensitive method (immunohistochemistry) to detect the BRAF^V600E^ phenotype the age difference to wildtype-tumors, especially in nevi, became smaller – an effect already shown before by Zalaudek et al. [28]. An explanation might be that, of coexisting subclones in melanocytic tumors [38], those harboring BRAF^V600E^ are more likely to have undergone senescence or regression [44,45] in older patients and are therefore less likely to be detected by methods with lower sensitivity (e.g. Sanger sequencing).

A limitation of our study is that it is difficult to differentiate “severe junctional dysplasia” from melanoma in situ and that this generates a bias towards selection of melanomas that are associated with banal or “moderately dysplastic” nevi. However, according to our experience, the term “severely dysplastic nevi” expresses diagnostic uncertainty and not biologic uncertainty. If the concept of “dysplasia” is based on the impossibility to differentiate “severe junctional dysplasia” of a nevus from “melanoma in situ” it immunizes itself against falsification, making it a problematic concept from a scientific point of view.

In sum we show that the presence of a BRAF- or NRAS mutation, at least in the present series, is not biologically relevant in the development of melanomas that arise in association with a nevus. Other, currently unidentified, genetic or epigenetic changes may play a role in the transformation of nevi to melanomas.

## Materials and Methods

### Ethics statement

The study was approved by the local ethics committee of the Medical University of Vienna, Austria. The institutional ethics committee waived the need for written informed consent from those participants from whom only archived biopsy specimens were used in a retrospective manner (Ethics committee protocol 598/2010). Their private information and specimens were coded and not collected through an interaction or intervention with individuals. All other participants gave their written informed consent to participate in the study.

### Histologic assessment of melanoma-associated nevi and control nevi

Criteria for the presence of a melanoma-associated nevus were used as published before by Stolz et al. [46]. All nevi were rated for the presence of different postulated morphologic features of “dysplastic” nevi [34] with the following definitions: *Bridging*: Nests of melanocytes within the epidermis spanning two or more rete ridges. *Lentiginous or epitheloid cell proliferation*: Melanocytes, arranged as variably sized nests and single cells within the epidermis. *Cytologicatypia* : Presence of enlarged nuclei, hyperchromatic nuclei and/or prominent eosinophilic nucleoli. *Fibroplasia*: Increased number of fibroblasts and collagen fibres arranged in a linear fashion around rete ridges beneath the epidermal part of the neoplasm. Fibroplasia around dermal nests of melanocytes was disregarded.

### Laser microdissection

For laser-capture-microdissection one or multiple 5µm-thick sections of the specimen were cut onto membrane slides and stained with Haematoxylin and Eosin. The respective tumor areas were cut using the MMI CellCut Plus - Laser Capture Microdissection Microscope (Molecular Machines & Industries, Switzerland). Figure S1.

### BRAF Exon 15 and NRAS Exon 2 sequencing

Tissue samples were treated using the QuickExtract^TM^ FFPE DNA Extraction Kit using standard protocols. Primers used for PCR-amplification of the BRAF Exon 15 were forward: 5’-TCATAATGCTTGCTCTGATAGGA-3’ and reverse: 5’-GGCCAAAAATTTAATCAGTGGA-3’, or forward (shorter amplicon): 5’-TGTTTTCCTTTACTTACTACACCTC-3’ and reverse: 5’-TAATCAGTGGAAAAATAGCCTC-3’. PCR-Primers for NRAS Exon 2 were forward: 5’-GATTCTTACAGAAAACAAGTG-3’ and reverse: 5’-ATGACTTGCTATTATTGATGG-3’. Successful amplification of the respective region was confirmed by visualizing 5µl of the PCR products on a 2% Agarose Gel containing GelRed™ Nucleic Acid Gel Stain. PCR products were cleaned up using USB® ExoSAP-IT® PCR Product Cleanup (Affymetrix®, Santa Clara CA), Sanger-sequencing was performed using the respective primers used in PCR.

### Immunohistochemical detection of BRAF^V600E^ mutation

Available tumor tissue was stained using the BRAF^V600E^-mutation specific antibody VE1 as published before [47]. VE1 antibody was kindly provided by Prof. Andreas von Deimling (University of Heidelberg). Every slide was evaluated by two observers (PT, HK), blinded to the sequencing result, in correspondence with a standard H&E-stained slide of the same tumor. Positive staining intensity of tumor tissue was rated semiquantitatively (“weak”, “intermediate” and “strong”, Figure S5). Conventional Sanger sequencing has been described to be inferior to BRAF^V600E^-specific immunohistochemistry by VE1 [47] in terms of sensitivity and specificity, therefore, in discordant cases mutation status as detected by immunohistochemistry was used for further analyses (BRAF^V600E^ mutation was detected by IHC in five melanomas and one associated nevus not previously found by Sanger-sequencing).

### Statistical analysis

To compare groups the McNemar-test (paired data), Chi-square (unpaired categorical/nominal data), Fisher’s-exact (smaller sample sizes), Wilcoxon signed-rank (paired continuous/nominal data) and T-test (unpaired continuous data; two-tailed p-value) was used. Multivariate comparison of characteristics between melanoma associated nevi and control nevi was performed using a forward conditional logistic regression model. As the presence of a junctional component was highly codependent (data not shown) to all measured histopathologic characteristics, two models were calculated: One excluding a junctional component as a predictor variable, one excluding all histomorphologic features.

All given P-values are two-tailed and a p-value <0.05 was considered significant. All statistical analyses were performed using IBM® SPSS® statistics Version 20.0.

## Supporting Information

Figure S1 Tissue as imaged with the laser-capture microdissecting microscope before (A) and after (B) microdissection and the corresponding collected cuts (C).
(TIF)

Figure S2 Images of a melanoma associated with a preexisting nevus. A - Clinical image; C - Dermatoscopic image; B&D-F - Histologic overview (H&E-staining); H&E-staining (G) and VE1-Immunohistochemitry (H) of the associated nevus; H&E-staining (J) and VE1-Immunohistochemitry (I) of the melanoma
(TIF)

Figure S3 Images of a melanoma associated with a preexisting nevus. A - Clinical image; C - Dermatoscopic image; B&D-F - Histologic overview (H&E-staining); H&E-staining (G) and VE1-Immunohistochemitry (H) of the associated nevus; H&E-staining (J) and VE1-Immunohistochemitry (I) of the melanoma
(TIF)

Figure S4 Overview of NRAS^Q61^ (**blue**) and BRAF^V600E^ (**red**) mutations in evaluated tumor material. Columns depict corresponding tumor parts.
(EPS)

Figure S5 Semiquantitative rating of VE1 - Immunohistochemistry. - Staining of nuclei is stronger ("weak"), equal ("intermediate") or weaker ("strong") than VE1-staining.
(TIF)
