# Supplementary figures and images for: NRAS and BRAF Mutations in Melanoma-Associated Nevi and Uninvolved Nevi
Source: PLoS One. 2013 Jul 8;8(7):e69639. doi: 10.1371/journal.pone.0069639 (PMC3704624; doi:10.1371/journal.pone.0069639)

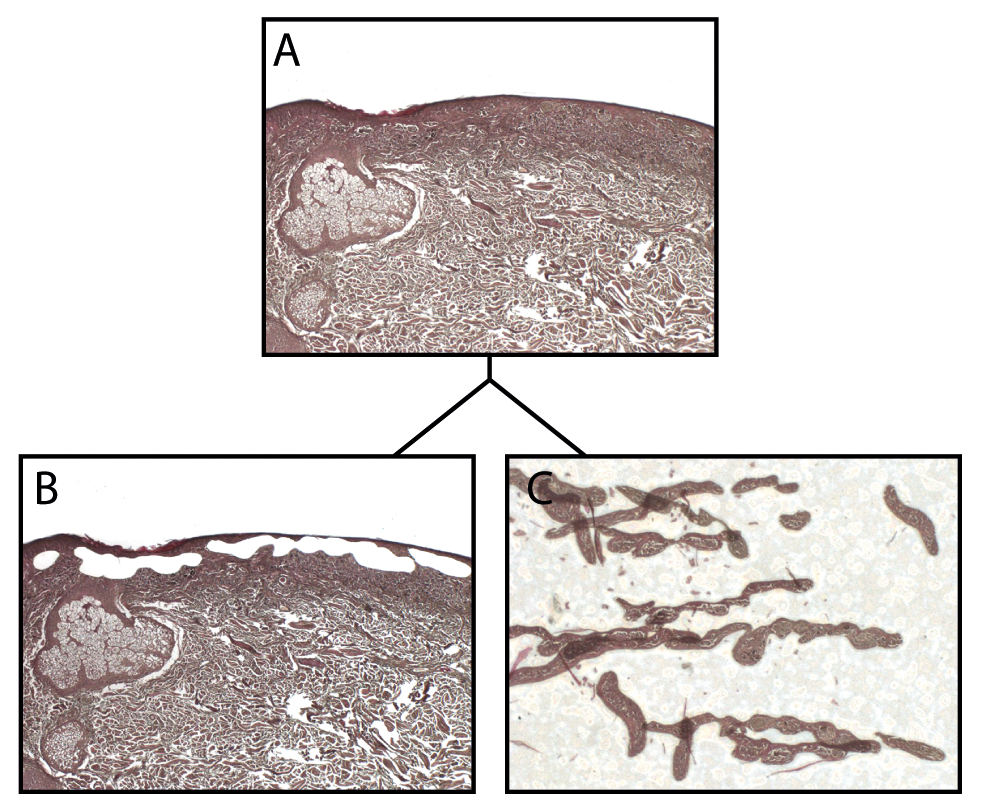

Supplement: Figure S1 — Tissue as imaged with the laser-capture microdissecting microscope before (A) and after (B) microdissection and the corresponding collected cuts (C). (TIF) [file pone.0069639.s001.tif]

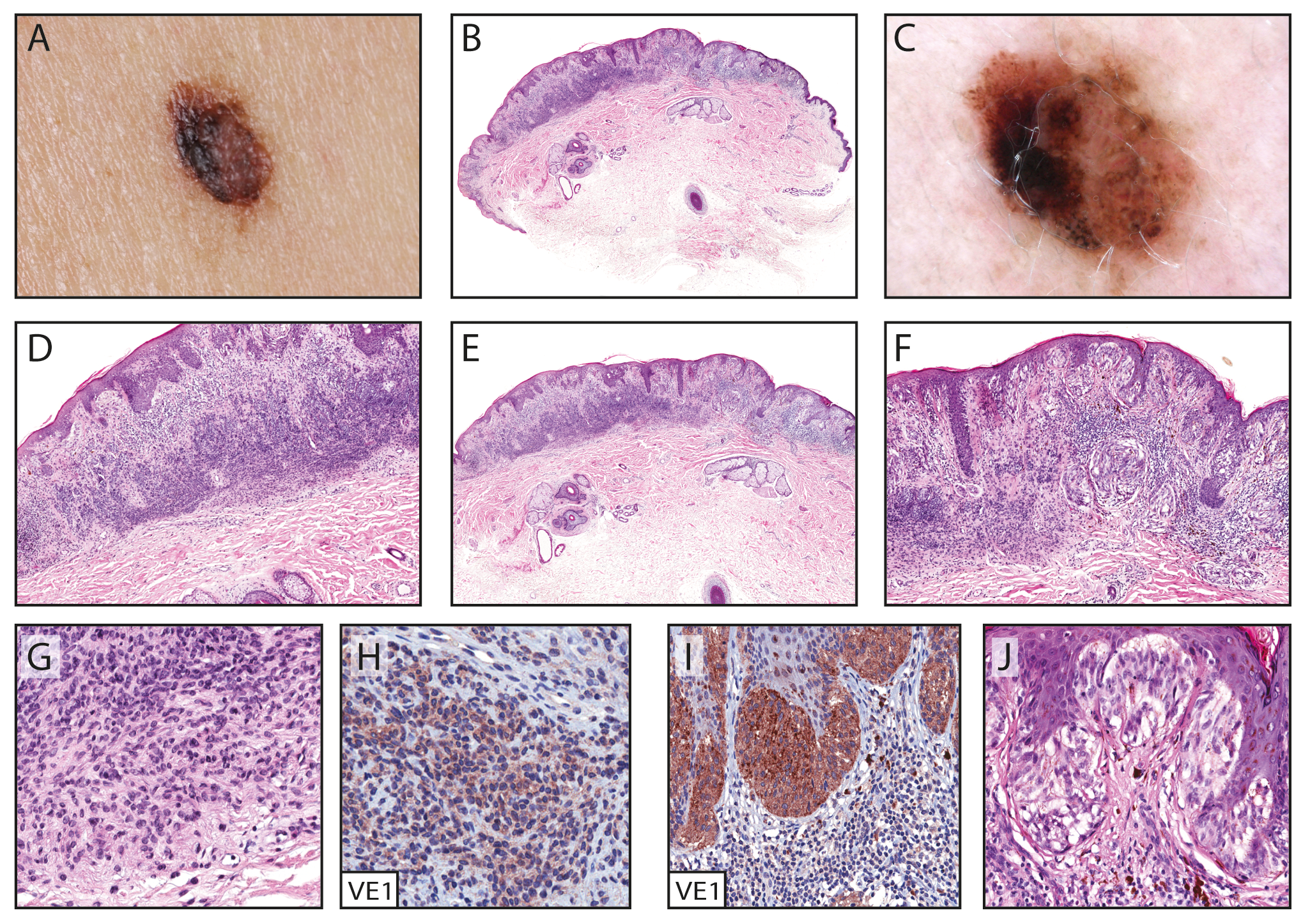

Supplement: Figure S2 — A - Clinical image; C - Dermatoscopic image; B&D-F - Histologic overview (H&E-staining); H&E-staining (G) and VE1-Immunohistochemitry (H) of the associated nevus; H&E-staining (J) and VE1-Immunohistochemitry (I) of the melanoma (TIF) [file pone.0069639.s002.tif]

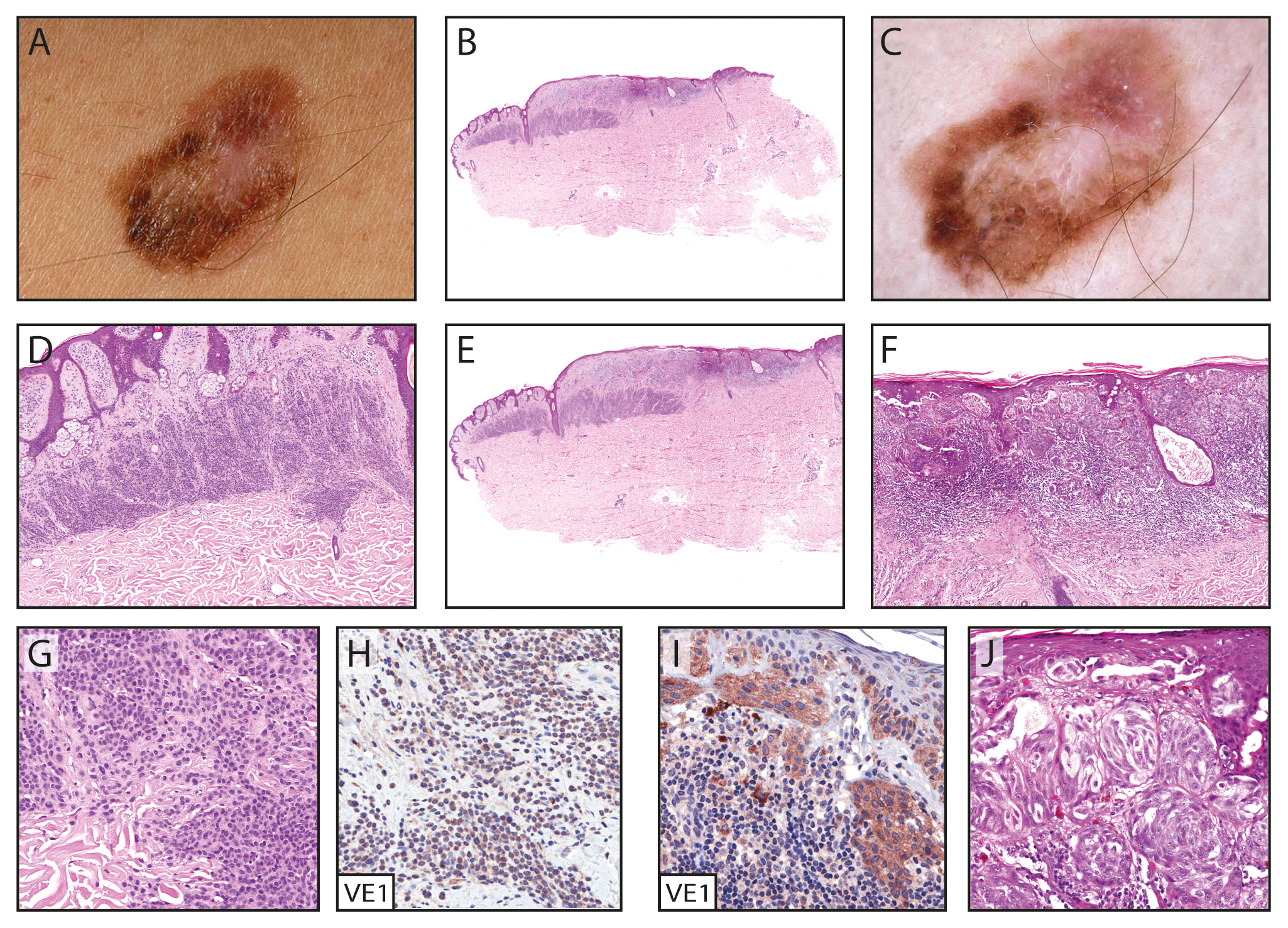

Supplement: Figure S3 — A - Clinical image; C - Dermatoscopic image; B&D-F - Histologic overview (H&E-staining); H&E-staining (G) and VE1-Immunohistochemitry (H) of the associated nevus; H&E-staining (J) and VE1-Immunohistochemitry (I) of the melanoma (TIF) [file pone.0069639.s003.tif]

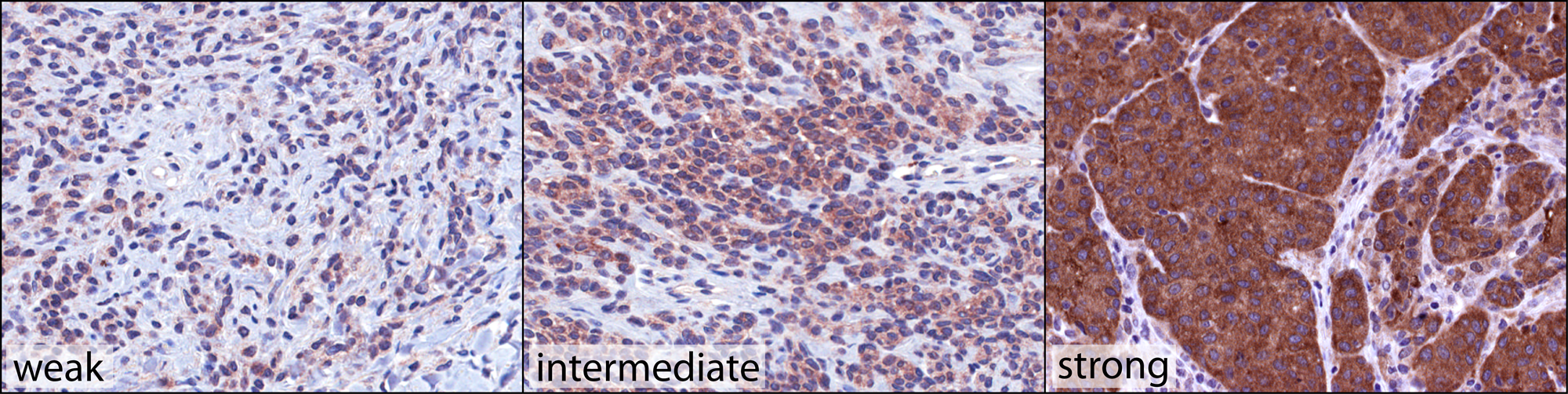

Supplement: Figure S5 — - Staining of nuclei is stronger ("weak"), equal ("intermediate") or weaker ("strong") than VE1-staining. (TIF) [file pone.0069639.s005.tif]
